# Machine Learning Models Predicting Solubility and Polymerizability of Polyimides Considering Multiple Monomers for CO_2_ Separation Membranes

**DOI:** 10.1002/minf.70032

**Published:** 2026-04-22

**Authors:** Yuto Shino, Motosuke Katayama, Yuri Ito, Hiromasa Kaneko

**Affiliations:** ^1^ Department of Applied Chemistry School of Science and Technology Meiji University Kawasaki Kanagawa Japan; ^2^ Nitto Denko Corporation Ibaraki Osaka Japan

**Keywords:** cheminformatics, copolymerization, machine learning, membranes, polymers

## Abstract

Membrane technologies for the separation of gases, such as CO_2_/CH_4_ mixtures, have attracted attention because of their high energy efficiency. Polyimides are considered promising membrane materials for CO_2_ separation, and there is a growing demand for materials with even higher performance. In the screening of candidate materials, it is essential to consider not only separation performance but also solubility and polymerizability during the synthesis process. Low solubility or polymerizability can inhibit membrane fabrication and the evaluation of separation performance, potentially leading to wasted resources and effort. In this study, we developed machine learning models to predict the solubility and polymerizability of polyimides. Mixture features derived from molecular descriptors of multiple monomers and mixing ratios were used as inputs for the classification models. The models were then applied to novel candidates, and their effectiveness was validated experimentally.

## Graphical abstract

We developed machine learning models to predict the solubility and polymerizability of polyimides for CO_2_ separation membranes. Mixture features derived from monomer descriptors and composition ratios enabled classification of synthesizability. Experimental validation demonstrated that this approach is promising for streamlining material screening and reducing unsuccessful synthesis attempts.

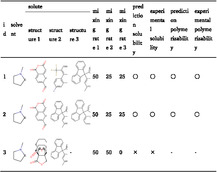

## 1 Introduction

Polymeric materials are essential to numerous modern industrial sectors, and considerable effort has been devoted to the development of high‐performance polymers. One such research effort involves the development of CO_2_/CH_4_ separation membranes utilizing polyimides. These membranes are designed to separate CO_2_ from CO_2_/CH_4_ gas mixtures, enabling the purification of CH_4_. Membrane‐based gas separation technologies offer advantages such as high energy efficiency, low cost, and excellent scalability [1]. Although polyimides have demonstrated significant potential as materials for CO_2_ separation membranes, the exploration of novel structures with improved separation performance remains an active area of research [2, 3]. In the screening of materials, not only separation performance (i.e., permeability and selectivity) but also solubility and polymerizability during synthesis must be considered. Polyimides are typically synthesized via polymerization in N‐methyl‐2‐pyrrolidone, but when the monomers or intermediates exhibit poor solubility in the solvent, precipitation can occur during the reaction, and the precipitate often does not redissolve [4, 5]. As a result, membrane formation and subsequent evaluation of separation performance become unfeasible. Furthermore, even with sufficient solubility, poor polymerizability can lead to low‐molecular‐weight polymers, which fail to provide the necessary mechanical strength for membrane fabrication, thereby hindering performance evaluation. These challenges may result in wasted resources and effort, diverting attention from the primary goal of improving separation performance. Therefore, the ability to predict the polymerizability and solubility of polyimides in advance is crucial for the efficient development of new materials [6].

Machine learning is a promising tool to address these challenges. For instance, classification models can be constructed to predict solubility and polymerizability based on chemical structures. In such models, molecular descriptors derived from chemical structures are used as input features X, and binary labels Y indicating the presence or absence of solubility and polymerizability are used to train Y = f(X).

Machine learning‐based approaches have primarily been developed for single‐component compounds; however, recent studies have begun to extend these methods to multicomponent systems [5, 7, 8, 9]. In previous work, descriptors for multicomponent systems have often been represented using composition‐weighted averages [8, 10, 11, 12]. This approach is effective for additive features such as molecular weight or the number of substituents. However, for features that are more strongly influenced by complex mixing effects, weighted averages may fail to capture interactions accurately, presenting challenges in representing mixture descriptors [13]. Moreover, in the context of polymer design, although copolymers can be regarded as mixtures of multiple monomers, research leveraging this representation remains limited [7, 10].

In this study, we predict the solubility and polymerizability of polyimides by calculating a variety of mixture descriptors that account for complex mixing effects. We used mixture descriptors, calculated from molecular descriptors and mixing ratios of multiple monomers including dicarboxylic anhydrides and diamines, as X and binary labels representing solubility and polymerizability as Y. Given that the mixture descriptors calculated from structural information are high‐dimensional and may include irrelevant features, the Boruta algorithm [14] was employed for feature selection. Based on X and Y, a classification model was constructed and used to predict the solubility and polymerizability of test data not included in the training set. Furthermore, the model was applied to predict the solubility and polymerizability of new candidate materials, and the predictions were experimentally validated to assess effectiveness of the models.

## 2 Method

### 2.1 Dataset

In this study, binary data were classified based on the presence or absence of polymerizability and solubility of polyimides. The dataset consisted of the SMILES representations of the solvent, multiple types of dicarboxylic anhydrides and diamines as solutes, and their mixing ratios. It included 85 samples labeled for solubility and 48 samples labeled for polymerizability.

### 2.2 Molecular Descriptors

Molecular descriptors are widely used as the input features X to develop quantitative structure–activity and structure–property relationship models employing various statistical methods. In this study, molecular descriptors calculated by RDKit and CODESSA software and chemical structural similarities between solutes and solvents were employed.

RDKit [15] is an open‐source library available for Python that calculates over 200 2D structural descriptors. CODESSA [16] computes more than 450 molecular descriptors using quantum chemical calculations provided by AMPAC. The descriptors calculated by CODESSA can be classified into constitutional, topological, geometrical, electrostatic, quantum chemical, and thermodynamic categories.

The similarity value used in this study represents the structural similarity between solutes and solvents and ranges from 0 to 1, with values closer to 1 indicating greater structural similarity. These similarity scores were computed by combining molecular fingerprints with similarity coefficients [17].Specifically, seven types of similarity coefficients and 13 types of molecular fingerprints, listed in Table 1, were employed for the calculations. The fingerprints used are as follows:

**TABLE 1:** Similarity coefficients.

Name	Measurement
Cosine [16]	$\frac{a}{\sqrt{\left(a+b)(a+c\right)}}$
Dice [18]	$\frac{2a}{2a+b+c}$
Hamming [19]	$\frac{a+d}{a+b+c+d}$
Russel–Rao [20]	$\frac{a}{a+b+c+d}$
Rogers–Tanimoto [21]	$\frac{a+d}{a+d+2(b+c)}$
Sokal–Sneath [22]	$\frac{a}{a+2(b+c)}$
Tanimoto [23]	$\frac{a}{a+b+c}$

1. MACCS Keys [24]

2. Atom‐pair [25]

3. RDKit [15]

4. Extended‐connectivity Fingerprint (ECFP0, ECFP2, ECFP4, ECFP6) [26]

5. Functional‐class Fingerprint (FCFP0, FCFP2, FCFP4, FCFP6) [26]

6. Topological‐torsion [27]

7. Avalon [28]

A total of 91 similarity descriptors were obtained from these combinations. Fingerprints were computed using RDKit [15], and similarity scores were calculated using scikit‐learn [29]. Hansen solubility parameters in practice (HSPiP) [30] was also used; HSPiP is a software tool based on Hansen solubility parameters that can be used to estimate molecular solubility parameters (δD, δ*P*, δ*H*) and **≈**60 other physicochemical properties using experimental and structural data.

### 2.3 Descriptors of Mixture

The dataset used in this study includes multiple types of dianhydrides and diamines along with their mixing ratios. To account for the complex effects of such mixtures, various types of mixture descriptors were calculated. As shown in Table 2, six calculation methods were employed: weighted average based on mixing ratio, weighted variance, geometric mean, harmonic mean, max pooling, and min pooling. These approaches allow for more diverse representations of the relationships between the features of each monomer, enabling a multifaceted evaluation of the structural characteristics of multiple solutes.

**TABLE 2:** Descriptors of mixture.

Name	Measurement
Weighted average	$x_{\mathrm{ave}}=\sum_{i=1}^{n}w_{i}x_{i}$
Weighted variance	$x_{\mathrm{var}}=\sum_{i=1}^{n}w_{i}{\left(x_{i}-x_{\mathrm{ave}}\right)}^{2}$
Geometric mean	$x_{\text{gmean}}={\left(\prod_{i=1}^{n}x_{i}\right)}^{\frac{1}{n}}$
Harmonic mean	$x_{\text{hmean}}=\left(\sum_{i=1}^{n}w_{i}\right)/\left(\sum_{i=1}^{n}\frac{w_{i}}{x_{i}}\right)$
Max‐pooling	$x_{\max}=\max(x_{1},x_{2},\ldots )$
Min‐pooling	$x_{\min}=\min(x_{1},x_{2},\ldots )$

### 2.4 Boruta Algorithm

The structural descriptors used in this study are high‐dimensional and may contain many irrelevant features. Furthermore, a large number of mixture descriptors were calculated, resulting in a substantial number of total features. The Boruta algorithm was employed for feature selection. The Boruta algorithm is a feature selection method based on feature importance in random forests (RFs). Shuffled versions of the original features—referred to as shadow features—are created, and feature importance scores are calculated. These shadow features are then compared with the original features, and only the original features with importance scores exceeding the top p% of the shadow features are counted in each iteration. This process is repeated, and the truly relevant features for explaining the response variable Y are identified using a two‐sided test.

The Boruta algorithm was implemented using the BorutaPy [31] package, with the parameter p set to 80, 90, and 100, and these results were compared.

### 2.5 Comparison of Explanatory Variables

To construct models for predicting polymerizability and solubility, 13 types of explanatory variable sets, as summarized in Table 3, were used. x1 includes descriptors calculated using RDKit, x2 consists of similarity indices, x3 is based on descriptors from HSPiP, and x4 includes descriptors from CODESSA. x5–x7 represent combinations of the aforementioned sets. x8–x10 were obtained by applying the Boruta algorithm to x7 for feature selection, while x11–x13 were generated by applying Boruta to x4 (CODESSA). Descriptors with RDKit and CODESSA were calculated separately for both solutes and solvents.

**TABLE 3:** Explanatory variables of x1–x13.

	Explanatory variables
x1	RDKit
x2	Similarity
x3	HSPiP
x4	CODESSA
x5	RDKit + similarity
x6	RDKit + similarity + HSPiP
x7	RDKit + similarity + HSPiP + CODESSA
x8	x7 + Boruta(*p* = 80)
x9	x7 + Boruta(*p* = 90)
x10	x7 + Boruta(*p* = 100)
x11	CODESSA + Boruta(*p* = 80)
x12	CODESSA + Boruta(*p* = 90)
x13	CODESSA + Boruta(*p* = 100)

## 3 Results and Discussion

The classification models used for model construction are listed below.

Linear classifier

1. LR: Logistic Regression [32]

2. LDA: Linear Discriminant Analysis [33]

3. LSVM: Linear Support Vector Machine [34]

Nonlinear classifier

1. NB: Naive Bayes

2. kNN: k‐Nearest Neighbors

3. NSVM: Nonlinear Support Vector Machine (Gaussian kernel) [34, 35]

4. DT: Decision Tree [36]

5. RF: Random Forest [37]

6. LGBM: Light Gradient Boosting Machine [38]

7. XGB: Extreme Gradient Boosting [39]

8. GBDT: Gradient Boosting Decision Trees [36]

To evaluate the constructed models, we employed double cross‐validation using the leave one group out (LOGO‐DCV) method. In LOGO‐DCV, the dataset is split according to each unique combination of solvent and solute, with one group used as the test set and the remaining groups used for training. For example, a group may contain data with the same solvent–solute pair but with different mixing ratios. The training data were further divided to perform fivefold cross‐validation for hyperparameter tuning. With the optimal hyperparameters, the model was trained on the training data and evaluated on the test group. This process was repeated so that each group served as the test set once. This approach was adopted to evaluate the predictive performance for novel combinations of solvent and solute.

### 3.1 Model Construction for Predicting Solubility

The predictive models for solubility were constructed using the variable sets x1–x13, as described in Section 2.5. Table 4 summarizes the best‐performing classification method for each explanatory variable set.

**TABLE 4:** Prediction accuracy of LOGO‐DCV for solubility classification.

	Method	Accuracy rate
x1	kNN	0.776
x2	LR	0.812
x3	RF	0.765
x4	LR	0.765
x5	LR	0.824
x6	LR, LSVM	0.824
x7	LR	0.800
x8	NSVM	0.859
x9	RF, LGBM	0.859
x10	RF	0.835
x11	LR	0.894
**x12**	**NSVM**	**0.929**
x13	RF	0.882

The combination of x12 as the explanatory variable set and NSVM as the classification algorithm yielded the highest classification accuracy of 0.929, demonstrating excellent predictive performance. Overall, the results indicate that applying feature selection using the Boruta algorithm improved prediction accuracy for solubility. This improvement can be attributed to the elimination of irrelevant or noisy features from the high‐dimensional descriptors, allowing the model to focus on the most informative variables. Table 5 presents the confusion matrix for the best‐performing model, showing that both positive (soluble) and negative (insoluble) samples were predicted without significant bias.

**TABLE 5:** Confusion matrix of the best‐performing method for solubility prediction.

	Positive, prediction	Negative, prediction
Positive	46	2
Negative	4	33

### 3.2 Model Construction for Predicting Polymerizability

The predictive models for polymerizability were constructed using the variable sets x1 to ×13. Table 6 presents the best‐performing classification method for each explanatory variable set. High prediction accuracy was obtained for most variable patterns. The results also confirmed that the use of the Boruta algorithm improved prediction performance, similar to the solubility prediction task. Because the dataset was imbalanced (44 positive and 4 negative samples), model performance was evaluated both accuracy and balanced accuracy. As shown in Table 6, the best‐performing model achieved high values for both metrics, indicating robust classification performance under class imbalance. Table 7 shows the confusion matrix for the best‐performing model, confirming that both polymerizable and nonpolymerizable samples were correctly identified.

**TABLE 6:** Prediction accuracy of LOGO‐DCV for polymerizability classification.

	Method	Accuracy rate	Balanced accuracy
x1	kNN, LSVM, NSVM	0.979	0.875
x2	DT	0.979	0.875
x3	LR, kNN, LGBM	0.958	0.750
x4	kNN	0.979	0.875
x5	kNN, NSVM	0.979	0.875
x6	kNN, NSVM	0.979	0.875
x7	kNN	0.979	0.875
x8	LR, kNN, LSVM, NSVM, RF	0.979	0.875
x9	LR, kNN, LSVM, NSVM	0.979	0.875
x10	LR, LSVM, NSVM	0.979	0.875
x11	LR, kNN, LSVM, NSVM, RF	0.979	0.875
x12	LR, kNN, LSVM, NSVM, RF	0.979	0.875
x13	kNN	0.958	0.864

**TABLE 7:** Confusion matrix of the best‐performing method for polymerizability prediction.

	Positive, prediction	Negative, prediction
Positive	44	0
Negative	1	3

### 3.3 Experimental Validation of New Material Candidates

High prediction accuracy was confirmed for both the solubility and polymerizability models. To further validate their performance, experimental verification was conducted using new material candidates. Table 8 summarizes the molecular structures of the solvents and solutes, along with their mixing ratios, used in seven experimental cases. Table 9 presents the predicted and experimental results for solubility and polymerizability for each combination. The solubility predictions matched the experimental results in 5 out of 7 cases, and the polymerizability predictions matched in 2 out of 3 cases. These results demonstrate that the proposed models achieve high predictive accuracy in practical scenarios. Consequently, the proposed approach is considered effective for estimating solubility and polymerizability of novel material candidates in advance.

**TABLE 8:** Solvent and solute structures and their mixing ratios in experimental verifications.

Id	Solvent	Solute			Mixing rate 1	Mixing rate 2	Mixing rate 3
		Structure 1	Structure 2	Structure 3			
1	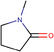	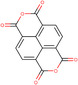	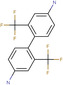	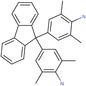	50	25	25
2	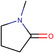	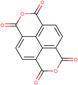	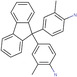	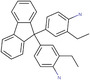	50	25	25
3	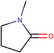	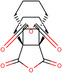	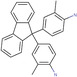	–	50	50	0
4	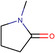	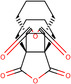	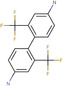	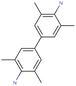	50	25	25
5	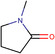	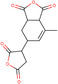	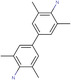	–	50	50	0
6	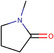	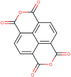	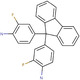	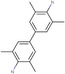	50	25	25
7	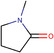	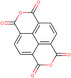	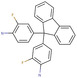	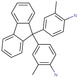	50	25	25

**TABLE 9:** Comparison of predicted and experimental solubility and polymerizability for tested samples.

Id	Prediction solubility	Experimental solubility	Prediction polymerizability	Experimental polymerizability
1	○	○	○	○
2	○	○	○	○
3	×	×	—	—
4	×	×	—	—
5	○	○	○	×
6	○	×	—	—
7	○	×	—	—

However, discrepancies were observed in the solubility predictions for No. 6 and No. 7. Both cases involve monomers containing multiple fluorine (F) substituents and cardo structures. While polyimides derived from highly fluorinated monomers are generally more soluble, those incorporating cardo structures may exhibit poor solubility depending on the number and position of the substituents. In these cases, it is presumed that the model predicted solubility based on the presence of fluorinated groups, whereas the actual insolubility may have been caused by the cardo moieties. These results suggest that further improvement of the model requires the inclusion of more diverse training data and the consideration of molecular descriptors that better capture such structural characteristics.

## 4 Conclusions

In this study, predictive models were developed to estimate the solubility and polymerizability of polyimides. To represent the complex structure of polyimides, a variety of mixture descriptors were calculated using structural descriptors of monomers and their composition ratios. The constructed models achieved high prediction accuracy for both solubility and polymerizability. Furthermore, the use of the Boruta algorithm led to an overall improvement in prediction performance by selecting important features and eliminating irrelevant ones. Experimental validation was conducted on several new candidate materials predicted by the model, and a high level of agreement between predictions and experimental results was confirmed. The proposed models were able to accurately predict the solubility and polymerizability of new candidate materials prior to synthesis, thereby facilitating the development of materials with desirable properties. Consequently, this approach is expected to reduce the number of required experiments, leading to decreased time and costs in the development of membrane materials.

## Funding

This work was supported by the Japan Society for the Promotion of Science (24K08152 and 24K01234).

## Conflicts of Interest

The authors declare no conflicts of interest.

## Data Availability

Research data are not shared.
